# 

**Published:** 1908-03-28

**Authors:** 


### King Edward VII. Sanatorium.

The first annual report of the King Edward VII. Sanatorium is in clearness and brevity an example to be followed. The institution has been full since March 1907. As elsewhere, patients are classified according to the extent rather than the severity of their pulmonary lesion. Full particulars as to diet and exercise are given, the Frimley system of graduated labour having been adopted at the instance of some members of the consulting staff. The effects of treatment can be readily appreciated, since synopses of each case on admission and on discharge are given, and may be compared. To some patients a considerable amount of daily exercise is allowed in spite of the existence of a little pyrexia—*e.g.* "eight miles and gardening," with a temperature range of 97.8°-100.4°. Others doing as much show only records like 97.2°-98.6°, which are generally considered the optimum limits. This is probably accounted for by the inevitable compromise confronting the sanatorium physician when he has to treat consumptives dependent for a livelihood on their own exertions, and with but a limited time at their disposal for treatment. A more apyretic state of the patients would unfortunately have been of better augury for their future. Tuberculous laryngeal disease was found in 11 per cent. of cases, and a feature of their treatment has been vocal rest. The laboratory results, including 500 opsonic indices taken on 40 patients, are not yet ripe for publication.

### BOOKS RECEIVED.

J. Wright and Company, Bristol.
"Experimental Prophylaxis of Syphilis." By Dr. Paul Maisonneave.

G. F. Hilcken, Librarian, Bethnal Green Free Library.
"The Christ in Shakspeare." By C. Ellis.

E. Goodman and Son, Taunton.
"Annual Reports for 1907 of the Sanitary Condition of the Urban and Rural Sanitary Districts of Taunton." By Henry J. Alford, M.D.

William Pile, Limited, Wallington.
"Health Report for 1907 of the Croydon Rural District by the Medical Officer of Health."

Henry Frowde and Hodder and Stoughton.
"Diets in Tuberculosis." By Noel D. Bardswell, M.D., and J. E. Chapman, M.R.C.S.

### Alcoholism in France.

While it is common in this country for us to compare ourselves unfavourably with other nations in the matter of temperance, our neighbours across the channel are beginning to lament their indulgence in alcohol. The French are a wine-drinking nation, but natural wine, such as they used to drink, contains but a small proportion of alcohol—often not more than one finds in so-called temperance drinks in this country. Perhaps during the years when the phylloxera ravaged the French vineyards a less pure wine was sold—one which owed its qualities to other chemistry than that of nature. But France raises no protest against the consumption of wine. It is not that which works havoc in its homes. One may place at the head of the causes of drunkenness in France absinthe—the popular drink in Paris, where it is said that 18,000 bottles are consumed daily. Absinthe is drunk by men, women, and even children in Paris. One observer noticed, not long since, "three boys, the eldest about twelve and the youngest hardly more than eight, seated outside a drinking-shop in one of the great avenues, each with his glass of absinthe before him, and his cigarette in his mouth." And no notice was taken of the sight, no comment by passers-by, no thought of prosecuting the publican for what was, nevertheless, a flagrant violation of the law.

At present, incredible as it may appear, France stands at the head of European nations in its consumption of alcohol. The amount stands at 14 litres of pure alcohol (of 100 degrees) per head of population per annum. This surpasses Belgium by four litres and Great Britain by five. In Paris there is a cabaret for every seventy-seven of the population, and foreign visitors are beginning to complain of the late hours and the noisy habits of those who frequent these places, which are found at almost every corner, even in the quieter streets. And not in Paris only is there an increase in drinking-shops. It is reckoned that within the last ten years these have increased in the provinces by 70,000. So serious is the matter that the Minister of Public Instruction has recently taken it up, and has appointed a special commission to inquire what is the best way to bring before the pupils in the elementary and secondary schools of France the dangers of alcoholism. A series of recommendations have been made, including the suggestion that suitable handbooks should be prepared for use in the different classes, in order to instruct the young in the nature and effects of alcohol, and that special lectures should be given by medical men each year on the subject.

Two societies have been formed to combat this national danger. One asks its members merely to pledge themselves to drink only light wines, cider, and beer, and these only at meals, never to enter a public-house, and not to taste those stronger drinks which are specially defined as alcoholic. This society is having considerable success among young people, including those in schools and colleges. The other is an absolutely total abstinence society, and is usually associated with definitely religious work. Nor has political action been wanting. The question of the injury wrought in the physique of the people by indulgence in alcohol was brought before a Commission on Hygiene which was ordered by the Chamber of Deputies. The question came before the commission whether the acknowledged danger due to indulgence in absinthe should be combated by total prohibition or simply by taxation of the liquor. M. Schmidt, the Deputy for St. Dié did his best to secure the prohibition of what he called the "green poison," but the commission finally decided to be content with taxation. The extent to which taxation will limit consumption remains to be seen. But the mere fact of the question being raised shows that the national conscience of France is aroused on the matter.

A second question also has arisen—that of adulteration. M. Villejean, Professor of Medicine and Chief Pharmacist of the Hôtel Dieu, who presided at the Commission on Hygiene, has given his support to the making of a distinction between "good" and "bad" absinthe. Naturally his action has given great annoyance to the extreme temperance party, who looked to him to support their cause. And on this side of the Channel there will be found but few who will distinguish between good and bad absinthe, for all are convinced of the insidious dangers of the liqueur. Nevertheless, people of moderate views will believe that a distinction between genuine and "faked" liquors is a helpful contribution to the discussion of any question of temperance.

### The 16th International Medical Congress.

International Prize for Ophthalmology.

Professor Emile Grósz, the Secretary-General, gives notice that the sixteenth International Medical Congress will be held at Buda-Pesth from August 29 to September 4, 1909, under the patronage of his Imperial and Apostolic Royal Majesty Francis Joseph I. The work of the Congress is divided into 22 divisions, with a special sub-section of orthopædics attached to the section of surgery. The Royal Hungarian Minister of the Interior offers a prize of one thousand crowns for the best essay on "The Ætiology of Trachoma." All works, whether in manuscript or published for the first time in 1907 or 1908, will be eligible for competition, provided they are in English, Hungarian, French, or German. Essays are to be sent to the Minister of the Interior (Belügy-ministeriun, Buda-Pesth Hungary) before December 31, 1908. The jury will be appointed by the Minister of the Interior, and the award will be made at the first meeting of the Congress. The subscription to the Congress is 25 crowns—about one pound sterling—the wives and daughters of members paying twelve and a half crowns. The officials of the English National Committee are the President (Dr. F. W. Pavy, F.R.S.) and the two Honorary Secretaries (Dr. Clive Rivière and Mr. D'Arcy Power). The address of the Honorary Secretaries is 10a Chandos Street, Cavendish Square, W.

### THE GRADUATES' MEDICAL SCHOOLS.

### Diary for the week March 30 to April 4.

**ROYAL COLLEGE OF PHYSICIANS, Pall Mall East.**

**At 5 p.m.** Lumleian Lectures (*continued*).

**March 31 and April 1,** Sir James Sawyer, M.D., **Points of Practice in Maladies of the Heart.**

**CENTRAL LONDON THROAT and EAR HOSPITAL Gray's Inn Road, W.C.**

**At 4 p.m.**

**April 2,** Dr. Wyatt Wingrave, **A Summary of Recent Pathological Experience in the Practice of the Hospital.**

**MEDICAL GRADUATES' COLLEGE AND POLYCLINIC, Chenies Street, W.C.**

**At 5.15 p.m.**

**March 30 and April 1,** Dr. C. O. Hawthorne, **The Clinical Anatomy and Physiology of the Nervous System.**

**March 31,** Mr. Henry Curtis, **Some Interesting Cases of Cranial Surgery.**

**April 2,** Mr. Gilbert Barling, **The Diagnosis and Treatment of Renal Mobility.**

**THE POST-GRADUATE COLLEGE, West London Hospital, Hammersmith, W.**

**At noon.**

**March 30,** Dr. Low, **Pathological Demonstration.**

**March 31 and April 2,** Dr. Pritchard, **Practical Medicine.**

**At 5 p.m.**

**March 31,** Mr. Lloyd Williams, **When to Extract—(*a*) Caries, Septic Conditions; (*b*) Overcrowding, Malocclusion; (*c*) Instruments and Methods.**

**April 1,** Dr. Beddard, **Practical Medicine—VI.**

**NORTH-EAST LONDON POST-GRADUATE COLLEGE, Prince of Wales's General Hospital, Tottenham, N.**

**At 4.30 p.m.**

**March 31,** Dr. Giles, **Pelvic Displacements.**

**LONDON SCHOOL OF CLINICAL MEDICINE, Seamen's Hospital, Greenwich.**

**At 2.15 p.m.**

**March 31,** Dr. Hewlett.

